# The impact of operating room noise levels on stress and work efficiency of the operating room team: A protocol for systematic review and meta-analysis

**DOI:** 10.1097/MD.0000000000028572

**Published:** 2022-01-21

**Authors:** Li Peng, Jia Chen, Hong Jiang

**Affiliations:** Department of Anesthesiology, Central Hospital of Enshi Tujia and Miao Autonomous Prefecture, Enshi, Hubei, China.

**Keywords:** meta-analysis, noise level, operating room, protocol, stress, work efficiency

## Abstract

**Background:**

There is no high-quality meta-analysis in the literature to determine the noise level in the operating room. Therefore, the aim of this study is to systematically review the available evidence in the literature to elucidate the impact of operating room noise levels on stress and work efficiency of the operating room team.

**Methods:**

Two individual researchers will conduct the platform searches on the PubMed, Cochrane Library, and Embase databases from inception to June 1, 2022. The cohort studies assessing the impact of operating room noise levels on stress and work efficiency of the operating room team will be included. The outcomes include total workload level, stress scores, anxiety scores, operation time. We will collect data according to the guidelines in the Cochrane Handbook for Systematic Reviews of Interventions. The Meta analysis will be performed using Review Manager version 5.3 provided by the Cochrane Collaboration. Risk bias analysis of the studies will be performed independently by two reviewers using the Cochrane Risk of Bias Assessment Tool.

**Results:**

The review will add to the existing literature by showing compelling evidence and improved guidance in clinic settings.

**Registration number:**

10.17605/OSF.IO/7N8RY.

## 1 Introduction

Environmental noise pollution is considered to be a common stressor. Nowadays, high noise levels in operating rooms are commonplace and often exceed the 30 dBA recommended threshold set by the World Health Organization.^[1,2]^ It was observed that noise pollution was mainly caused by employee-related behaviors and surgical equipment. Its adverse effects range from poor concentration to mental and physical stress. Continued exposure may also cause permanent noise-induced hearing loss and tinnitus in the staff working in the operating room.^[3,4]^

Dholakia et al prospectively assessed operating room noise levels and found a positive correlation between intraoperative noise levels and surgical site infection. They hypothesized that the inattention caused by high levels of noise may affect the ability of operating room staff to perform aseptic techniques, thereby increasing the possibility of surgical site infections.^[5]^ Intraoperative noise can affect the reasoning of operating room staff and their ability to perform tasks. For example, the misjudgment rate of the anesthesiologist may increase when the patient's condition changes in a noisy environment.^[6,7]^ Furthermore, intraoperative noise may affect effective communication between operating room staffs, and ineffective communication is a major contributor to adverse events. In addition to increased stress, increased fatigue among staff may be partly due to increased intraoperative noise levels.^[8–10]^

The above-mentioned hazards of noise levels to staff are based on only a few studies and theoretical reasoning. There is no high-quality meta-analysis in the literature to determine the noise level in the operating room. Therefore, the aim of this study is to systematically review the available evidence in the literature to elucidate the impact of operating room noise levels on stress and work efficiency of the operating room team.

## 2 Materials and methods

### 2.1 Search strategy

Since this study is on the basis of published or registered studies, ethical approval and informed consent of patients are not required. Two individual researchers will conduct the platform searches on the PubMed, Cochrane Library, and Embase databases from inception to 0June 1, 2022. Literature retrieving will be carried out through a combined searching of subject terms (“MeSH” on PubMed and “Emtree” on “Embase”) and free terms on the platforms of PubMed and Embase, and through keywords searching on platform of Cochrane Library. References within included articles will be reviewed to include articles that are not included within our literature search. There is no restriction on year of publication. The systematic review protocol has been registered on Open Science Framework registries. The registration number is 10.17605/OSF.IO/7N8RY.

### 2.2 Eligibility criteria

Included studies will be considered eligible if they met the Population, Intervention, Comparator, Outcomes, and Study design criteria as follows:

Population: operating room team including surgeons, nurses, and anesthetists;
Intervention: high-level noise group;
Comparator: low-level noise group;
Outcomes: total workload level, stress scores, anxiety scores, operation time;
Study design: cohort studies;
Exclusion criteria include review articles, letters, comments, studies without a control group, and studies with insufficient outcome data.

### 2.3 Data extraction

Data extraction will be performed by 2 independent reviewers using standardized data extraction forms. In studies where data is missing or unavailable for meta-analysis or where data is presented only graphically, an attempt will be made to contact the corresponding authors by e-mail. Otherwise, we will collect data according to the guidelines in the Cochrane Handbook for Systematic Reviews of Interventions. If necessary, the extraction of incomplete data will be abandoned. When disagreement in the collection of data occurrs, it will be resolved through discussion. We will extract the following information: general information for the articles including the first author, year, country, sample size; demographic characteristics including gender, age and other information including surgery type and American Society of Anesthesiologists classification; and outcome information including total workload level, stress scores, anxiety scores, operation time.

### 2.4 Data analysis

According to the basic characteristics of the included studies, the Meta analysis will be performed using Review Manager version 5.3 provided by the Cochrane Collaboration. Given the characteristics of the extracted data in the review, continuous outcomes will be expressed as the mean differences with 95% confidence intervals. Differences in categorical variables will be expressed as risk ratio values and 95% confidence intervals. Heterogeneity will be assessed by means of *I*^2^ statistics. *I*^2^ ≥ 50% represent high heterogeneity. A standardized mean difference will be used when the studies included in the meta-analysis assess the outcome based on different scales. Initially, a fixed-effect model will be used to compare the outcomes, unless the heterogeneity tests indicate that the *I*^2^ statistic ≥50% and substantial heterogeneity existed between studies; in this case, the reasons for this heterogeneity will be searched for and a random-effect model will be used for comparison. The publication bias will be assessed by using funnel plots diagram. The funnel plot asymmetry will be evaluated by an Egger linear regression test to reveal any possible publication bias. Sensitivity analyses will be undertaken to determine the potential source of heterogeneity when significant.

### 2.5 Risk of bias

Risk bias analysis of the studies will be performed independently by two reviewers using the Cochrane Risk of Bias Assessment Tool for the following criteria: random sequence generation, allocation concealment, blinding of outcome assessment, incomplete outcome data, selective reporting, and other bias. When reviewers have different opinions, consensus is reached through discussion. Evidence from research studies is ranked as having either “high,” “low,” or “unclear” risk of bias.

## 3 Discussion

The World Health Organization recommends that the level of continuous background noise in hospitals should not exceed 30 dB during the day to maintain speech intelligibility. Previous studies mainly focused on solely measuring decibel levels in the operating room, and several recent reviews explored this topic.^[11]^ There is no high-quality meta-analysis in the literature to determine the noise level in the operating room. Therefore, the aim of this study is to systematically review the available evidence in the literature to elucidate the impact of operating room noise levels on stress and work efficiency of the operating room team.

## Author contributions

**Conceptualization:** Li Peng.

**Data curation:** Li Peng, Jia Chen.

**Funding acquisition:** Hong Jiang.

**Investigation:** Li Peng, Jia Chen.

**Methodology:** Jia Chen.

**Project administration:** Hong Jiang.

**Resources:** Hong Jiang.

**Supervision:** Hong Jiang.

**Validation:** Jia Chen.

**Writing – original draft:** Li Peng, Jia Chen.

**Writing – review & editing:** Hong Jiang.
